# The Role of Social Movements in Reducing Harmful Corporate Practices: Comment on "National Public Health Surveillance of Corporations in Key Unhealthy Commodity Industries – A Scoping Review and Framework Synthesis"

**DOI:** 10.34172/ijhpm.8664

**Published:** 2024-11-10

**Authors:** Nicholas Freudenberg

**Affiliations:** School of Public Health, City University of New York, New York City, NY, USA.

**Keywords:** Commercial Determinants, Social Movements, Health Activism, Corporate Practices

## Abstract

Systematic public monitoring of the practices of corporations that harm health is a necessary but not sufficient measure to reduce the adverse impact of these practices. By supporting social movements and health activist campaigns that seek to modify the corporate structures, systems and practices that harm health, public health professionals and researchers can bring powerful new voices into this crucial public health task. Partnerships between the public health organizations and social movements and activists who seek to make human and planetary well-being more important objectives than higher corporate profits can help to achieve this aim. Public health professionals can play an important role in supporting such partnerships.

 A recent scoping review by Bennett et al concluded that “systematic monitoring of the practices of Unhealthy Commodities Industries is likely to enable governments to mitigate the negative health impact of corporate practices.”^1^ Based on this review, the authors recommended a framework to inform public health surveillance including identification of the key actors, corporate practices, and outcomes that could be monitored.

 This framework and review constitute an important contribution to improved surveillance of harmful corporate practices and build on previous literature.^2^ However, as Bennett et al acknowledge, evidence suggests that government monitoring of corporate practices is a necessary but insufficient step to achieve the goal of reducing the adverse consequences of these practices on human and planetary health.^3^ In this commentary, I suggest an additional approach that can contribute to this goal.

 Relying on government monitoring harmful practices is necessary but insufficient for reducing their harm for several reasons. First, few governments currently have the capacity or will to monitor these practices systematically.^2^ The methodological challenges, the complexity of the task, and opposition from powerful stakeholders are among the obstacles to establishing systematic monitoring systems.^4^

 Second, monitoring harmful practices can contribute to improved population health and reduced health inequities only if governments are willing to act on the findings from their monitoring systems. While governments vary in their ability and willingness to enforce environmental and health regulations, few governments have made reducing harmful business practices a public health priority, despite the evidence that it could contribute to improved health outcomes. Thus, the record to date suggests that the public health community will need to do more than propose more active monitoring to bring about meaningful reductions in the damage to global health that harmful corporate practices impose.

 Third, even when governments do monitor and regulate harmful corporate practices, their ability to stay focused on these tasks, or modify their monitoring and regulatory processes based on changing impact may be limited. Once again, pressure from corporate actors can discourage consistent attention or innovations in these processes. In addition election cycles in the United States, Brazil, the United Kingdom, the European Union Parliament, and elsewhere in the last decade show that politicians supporting or opposing more forceful regulation of corporations can be swept into or out of office, leading to rapid changes in regulatory regimes that have been established.^5^

 Several recent reviews summarize the evidence on these challenges to effective monitoring and regulation of harmful corporate practices.^5,6^ For public health professionals and researchers committed to reducing the harm from corporate practices, other approaches are needed. One such option is to support and strengthen the activities of social movements and health activist organizations that have already taken on commercial actors. In this commentary, I describe these forces and the strategies they have used to put pressure on governments, businesses, and the public to act to reduce commercial harm. I suggest some ways that the public health community can use its capacities and resources to support these social movements. Finally, I propose the creation of social movement/public health partnerships to pursue the shared goals of these two actors.

 Who are the social movements taking on commercial determinants of health, a term now commonly used to describe corporate practices that influence health? An early example was the tobacco control movement in which civil society groups and government partnered to enact and then monitor the Framework Convention on Tobacco Control.^7^ This agreement specified that the tobacco industry could not participate in setting government policy on tobacco.

 In the last few years, the climate justice movement has pressured government and international bodies to act more forcefully to require the fossil fuel industry and its partners to reduce carbon and greenhouse gas emissions and modify other political and business practices that contribute to the global climate emergency. The AIDS movement, the drug users harm reduction movement, and activists mobilized to respond more effectively to the COVID-19 pandemic have demanded that pharmaceutical companies value human health over it own profits and make more determined efforts to ensure that essential medicines and vaccines are available to all who need them and harmful drugs like OxyContin and other opioids are not inappropriately promoted. These groups have used litigation, public demonstrations, and civil disobedience, among other tactics, to force governments to act and to pressure drug companies to act more responsibly.^8^

 While no single definition fully captures the variety and diversity of social movements, several key characteristics define their capacity to take on commercial determinants of health. First, social movements act over time and sometimes across borders. In some cases, this enables activists to transcend the limited attention span or commitment to protecting the health of a particular regime or nation. The long-lasting campaign to Boycott Nestle illustrates the staying power of the movement to limit promotion of infant formula—as well as the challenges of taking on a global corporation.^9^

 Second, social movements help people articulate the grievances they experience in their daily lives and bring them into the political arena, to make the personal political, as the women’s movement has insisted. Activist campaigns enable individuals to translate the dissatisfaction that results from harm from employers, the food industry, mining corporations, chemical polluters, and predatory financial firms into political demands, thus putting pressure on governments, businesses, and investors to act to mitigate or prevent the harm.

 Third, social movements have experience using a variety of collaborative and contentious strategies to pressure governments and businesses to act more forcefully to reduce harmful corporate practices. Their diverse repertoires of action can convince government to act, strengthen the weak backbones of government officials, or focus media attention on health harming practices. In these ways, social movements, experienced working in situations of asymmetric power, can help to increase the capacity of both the public and government officials to reframe political struggles, win over new supporters, and transcend or bypass the sometime constrained and cumbersome processes governments use to advance desired policy goals.

 How can public health professionals support social movements to advance their efforts to reduce harmful corporate practices, systems, and structures? First, they can use their positions and influence with governments to educate policy-makers about the costs imposed by corporate determinants of health and the health and the economic benefits of reducing these harms. Second, they can evaluate government and business strategies to address harm and provide credible and accurate evidence to guide policy. Evidence from the tobacco, fossil fuel, alcohol, pharmaceutical, and food industries shows how the researchers they pay have distorted or falsified their findings to support their sponsors. Independent academics can play an important role in highlighting these practices and developing university rules that sanction those who publish deliberately misleading findings.^10^

 Finally, and perhaps most importantly, public health professionals can work with social movements to de-normalize the public acceptance of laws, values, and governance procedures that enable corporations to persist in their practices that harm human and planetary health. Writing about the tobacco control movement, Mahood wrote that the goal of denormalization is to “shift the focus from individual smokers’ judgment to corporate misbehavior showing how the industry has ‘operated outside the boundaries of civilized corporate behavior’ by marketing a deadly product.”^11^ Others have proposed that counter-marketing the harmful products and practices of the tobacco, food, alcohol industries, as well as the practices of banks and lending institutions can contribute to such denormalization.^12^ By supporting laws, regulations, and values that de-normalize deceptive and predatory marketing, misuse of science, tax avoidance and evasion, and corruption, public health professionals can assist in de-normalizing these practices.

 More pragmatically, public health professionals and scholars can assist social movement organizations and activists to synthesize the scholarship that informs their work, evaluate their policy and mobilization strategies, frame their public messages, and connect them to policy-makers. By using their institutional resources, professional capacities, and social networks to support measures and actions that social movements initiate to reduce harmful corporate practices, public health practitioners can contribute to another practice domain.

 Finally, public health professionals and scholars can join with social movements to create social movement/public health partnerships. Sustainable and innovative alliances between these two constituencies could support sympathetic elected officials and persuade undecided or skeptical ones. They could operate across academic, political, cultural, and media borders, giving a coherence to the opposition to the often more unified corporate voices. Such alliances could also constitute a counterweight to the influential public/corporate partnerships that sway business-friendly politicians.^13^ The Global Alliance for Tobacco Control, the Nestles boycott, and the Global Climate Health Alliance illustrate different approaches to this strategy.

 Others have suggested additional important roles that activists and social movements can play in advancing health and health equity in general and in building momentum for specific changes in the political and economic corporate systems, structures, and practices that harm health.^14,15^ By translating this emerging body of literature into specific roles and responsibilities and a policy agenda for public health professionals and researchers who seek to support social movements working to reduce corporate harms, the public health community can bring powerful new voices into the efforts to realize Bennett and colleagues’ call for improved surveillance of unhealthy corporate practices.

## Ethical issues

 Not applicable.

## Conflicts of interest

 Author declares that he has no conflicts of interest.
